# Supplementary material for: Boosting hot electron flux and catalytic activity at metal–oxide interfaces of PtCo bimetallic nanoparticles
Source: Nat Commun. 2018 Jun 8;9:2235. doi: 10.1038/s41467-018-04713-8 (PMC5993833; doi:10.1038/s41467-018-04713-8)
Supplement: Supplementary file 1 — Supplementary Information [file 41467_2018_4713_MOESM1_ESM.pdf]

Supplementary Information

Boosting hot electron flux and catalytic activity at metal–oxide interfaces of PtCo bimetallic nanoparticles

H. Lee et al.

Supplementary Methods

PtCo nanoparticle synthesis. To prepare the PtCo bimetallic nanoparticles (NPs) with well-controlled stoichiometry, we used a conventional polyol reduction method in which we co-reduced the two metal precursors and the initial concentration ratio of the cobalt-to-platinum precursors determined the final stoichiometry[1, 2]. The stoichiometric ratios of Co to Pt (*i.e.* Pt_3Co_1, Pt_1Co_1, and Pt_1Co_3) were controlled by the ratio of the cobalt-to-platinum precursor concentrations in the initial solution. For the Pt_1Co_1 NPs (1:1 ratio), 0.1 mmol of cobalt (II) acetylacetonate ($Co(acac)_2$, 26.6 mg, Sigma Aldrich 97% pure), 0.1 mmol of platinum (II) acetylacetonate ($Pt(acac)_2$, 40.6 mg, Sigma Aldrich 97% pure), and 55 mg of poly(vinylpryrrolidone) (PVP, Mw = 55,000) were dissolved in 5 mL of ethylene glycol (Sigma Aldrich) in a 50 mL 3-neck round-bottom flask. After adding the precursors, the reaction mixture was stirred under constant heating at 80 °C while the flask was evacuated for 30 min; the flask was then purged with Ar for 10 min. Afterwards, the flask was heated to 210 °C for 15 min and held at that temperature for 10 min with vigorous stirring. During heating, the color of the solution changed from orange to black at about 198 °C, indicating the formation of the colloidal PtCo nanoparticles. The reaction was then terminated by cooling to room temperature. The resulting Pt_1Co_1 NPs were isolated by precipitation using acetone and washed several times with a mixture of acetone and ethanol by centrifugation at 5000 rpm for 10 min. The as-synthesized NPs were re-dispersed in ethanol and stored until further use. For the other compositions (Pt_3Co_1 and Pt_1Co_3), the ratio of cobalt acetate to platinum acetate was modified while fixing the total precursor concentration at 0.2 mmol.

Co nanoparticle synthesis. For the pure Co NPs (*i.e.* Pt_0Co_1), 52.6 mg of $Co(acac)_2$ and 111 mg of PVP were dissolved in 8 mL of benzyl alcohol. After vacuum degassing, the temperature was increased to 150 °C with Ar flow and 2 mL of 1,6-hexanediol dissolved in benzyl alcohol (2M) was injected into the solution and the solution was kept at 150 °C for 20 h.

Pt nanoparticle synthesis. For the pure Pt NPs (*i.e.* Pt_1Co_0), 250 mg of $H_2PtCl_6{\cdot}6H_2O$ was used as the metal precursor, which was dissolved in 50 mL of ethylene glycol and 5mL of a 0.5 M NaOH solution and stirred at room temperature. After 20 min of stirring, the mixture was heated to 160 °C for 3 hr while bubbling N_2. The resulting Pt NPs were precipitated with 2 ml of 2 M HCl and dispersed in an ethanol solution containing 12.2 mg of PVP (Mw = 29,000). After cooling, we again followed the same purification procedure as that for the PtCo NPs (*i.e.* precipitation and washing with acetone and ethanol).

Characterization. We characterized the structure of the Pt_xCo_y NPs using transmission electron microscopy (TEM), high-angle annular dark-field scanning transmission electron microscopy (HAADF-STEM), and STEM-energy dispersive X-ray spectroscopy (STEM-EDS) (Supplementary Fig. 2 and 3). The chemical compositions of the Pt_xCo_y NPs were analyzed using both X-ray photoelectron spectroscopy (XPS) and inductively coupled plasma mass spectroscopy (ICP-MS) (Fig. 2d and Supplementary Fig. 4 and Table 1). For further microstructural examination of the Pt_xCo_y NPs, X-ray diffraction (XRD) was conducted for all NP samples (Fig. 2e) and the average crystallite sizes were calculated (Supplementary Table 2).

Supplementary Notes

Supplementary Note 1 | Estimation of Schottky barrier height. To study the conductive properties of the fabricated hybrid Au/TiO_2 nanodiode with Pt_xCo_y NPs, I–V curves were recorded (Fig. 1f). In thermionic emission theory, the current through a forward-biased metal–semiconductor Schottky diode is given by

$$I = AA^{*}T^{2}exp\left(-\frac{q\varphi_b}{k_BT}\right)exp\left(\frac{q(V-IR_s)}{\eta k_BT}\right), \qquad \text{(Supplementary Equation 1)}$$

where A is the diode area, A^{*} is the Richardson constant, T is the temperature, q is the elementary charge, k_B is the Boltzmann constant, φ_b is the Schottky barrier height, η is the ideality factor, and R_s is the series resistance of the diode. Supplementary Equation 1 cannot be used directly for fitting I–V curves because the dependence of the diode current on the bias voltage is expressed implicitly. Thus, we used the reverse function for fitting (Supplementary Equation 2), in which the voltage bias is given as a function of the diode current.

$$V = \frac{\eta k_BT}{q}ln\left(\frac{I}{I_0}\right) + IR_s \qquad \text{(Supplementary Equation 2)}$$

Here, $I_0 = AA^{*}T^{2}exp\left(-\frac{q\varphi_b}{k_BT}\right)$ denotes the saturation current. Fitting a typical I–V curve measured from the Au/TiO_2 nanodiode with Pt_xCo_y NPs to equation S2 resulted in $\varphi_b = 0.7$ eV, $\eta = 1.4$, and $R_s = 120\ \Omega$ (Fig. 1g).

Supplementary Note 2 | Hot electron detection using a catalytic nanodiode. Typically, an average activation energy of 11 kcal/mol is required to initiate the hydrogen oxidation reaction on the Pt surface in a thermally activated process. Therefore, we conducted current measurements on the Pt_xCo_y NPs/Au/TiO_2 catalytic nanodiodes in a H_2 (15 Torr) + O_2 (745 Torr) mixture while increasing the temperature from 30 to 120 °C to activate H_2 oxidation on the nanocatalysts. With sufficient reaction rates, a continuous flow of hot electrons induced by the exothermic reaction on the catalytic NPs could be detected as a current signal, or so-called chemicurrent. However, for accurate analysis of the flow of hot electrons, it is necessary to distinguish the chemicurrent (I_{ch}) from the thermoelectric current (I_{th}), which is caused by heat transfer along the surface of the nanodiode from the external heater. Therefore, we conducted additional current measurements under nonreactive conditions of pure O_2 (760 Torr) to eliminate the thermoelectric current from the total current (I_{tot}) measured from the nanodiode at these conditions, and thus obtained an accurate chemicurrent (*i.e.* $I_{ch} = I_{tot} - I_{th}$).

When chemical reactions occurred on the Pt_1Co_0, Pt_3Co_1, Pt_1Co_1, and Pt_1Co_3, the current signals measured in H_2+O_2 were larger than those detected in pure O_2 over the entire temperature range (Fig. 3a and Supplementary Fig. 5). However, in the case of the Pt_0Co_1 NPs, the thermoelectric current was close to the total current measured under the H_2+O_2 mixture, indicating that no chemical reaction occurred (Supplementary Fig. 5d). For more accurate measurements, we ensured that the electrical properties of the devices remained unchanged before and after operation by checking the I–V characteristics under the reaction conditions (Supplementary Fig. 6). We repeated the measurements three times for all samples and normalized the current signals with respect to catalytic NP coverage on the surface of the Au thin film.

Supplementary Note 3 | XPS analysis for defining the CoO/Pt interfacial area in PtCo NPs. There are numerous issues that contribute to modification of the electronic and chemical properties of bimetallic nanocatalysts, including surface segregation, ligand, geometry, and support effects as well as size, shape, and composition effects. Recent discussion indicates that the metal–oxide interface formed at the surface of bimetallic NPs plays a key role in determining catalytic performance. In previous studies using *in situ* X-ray adsorption and *in situ* TEM, the surface configuration of PtCo NPs changed when different gas environments were introduced[3, 4]. For example, under oxidizing environments, CoO segregated on the surface of NPs within a few seconds because the strength of the Co–O bond is much stronger than the Pt–O bond; whereas under reducing conditions, the Pt segregated back to the surface as the Co oxides were reduced.

Based on this, we analyzed the surface oxidation states of the PtCo NPs with XPS and found that the CoO is segregated on the surface of the PtCo NP after the H_2 oxidation reaction. The surface chemical composition and oxidation states of the Pt_xCo_y NPs were analyzed using XPS (K-alpha, Thermo VG Scientific) with a monochromatic Al–Kα X-ray source (1486.3 eV). All the samples were prepared as a 2D array on the SiO_2 substrate using the Langmuir–Blodgett technique. After survey scanning, regional scans for C 1s, O *1s*, Pt *4f*, Co *2p*, and valence band (VB) spectra were conducted with a 50 eV pass energy, 0.05 eV step size, and 50 ms dwell time (Supplementary Fig. 10–14). For charge compensation at the surface, a flood gun was used during all the scanning processes. All the XPS spectra were calibrated on the basis of the adventitious C 1s peak at 284.8 eV of binding energy and the inelastic backgrounds of all the spectra were subtracted using the Shirley background. For quantitative analysis of the composition, the peak area of each element (*i.e.* Co *2p*, Pt *4f*) was normalized with XPS sensitivity factors (12.6 for Co *2p* and 15.5 for Pt *4f*) and the atomic ratios for all the Pt_xCo_y NPs were estimated (Fig. 2d, Supplementary Fig. 3 and Table 1).

We also identified the existence of CoO (Co^{2+}) on the PtCo bimetallic NPs after H_2 oxidation. As shown in Supplementary Fig. 11, the Co *2p* peak spectra of the PtCo bimetallic NPs (*e.g.* Pt_3Co_1, Pt_1Co_1, Pt_1Co_3 NPs) are deconvoluted into three peaks corresponding to the metallic Co (Co^0) at ~ 777.7 eV, CoO (Co^{2+}) at ~ 781.1 eV, and a satellite peak at ~ 786.0 eV. The strong satellite peak in the Co 2p spectra clearly indicates that a substantial amount of Co in the PtCo bimetallic NPs is oxidized as a form of CoO rather than Co_3O_4. Even though Co_3O_4 is a more thermodynamically stable phase, a CoO layer on nanoparticles under oxidation conditions appears to form and be stable[3-6].

Particularly, in the case of the Pt_3Co_1 NPs, it was clearly confirmed that 23 % of the metallic Co was further oxidized and formed CoO on the surface of the NPs after the H_2 reaction than before the reaction (Fig. 4). However, Pt is rarely oxidized regardless of the composition and reaction, as shown in the deconvoluted Pt *4f* peaks of the PtCo bimetallic NPs, where the metallic Pt states (~ 72 at. %) are dominant over other oxide states, such as PtO (~ 22 at. %), PtO_2 (~ 6 at. %) (Supplementary Fig. 10 and 11).

We mathematically estimated the coverage of the CoO layer on the PtCo NPs with a hypothesis that the CoO exists as a monolayer. Surface atoms comprise approximately 45–50 % of the total atoms in the NP with a particle diameter of 2.5 nm. The relative ratio of CoO increased from 16.5 to 59.6 % as the Co content increased in the CoPt NPs (Fig. 4b); we obtained CoO coverage values of roughly 33, 78, and 119 % for Co_1Pt_3, Co_1Pt_1, and Co_1Pt_3, respectively. Therefore, we conclude that the deactivation of catalytic activity

originated from the reduced interfacial area of the CoO/Pt in the CoPt NPs as the Co content increased, which is consistent with the trends for conventional CO oxidation[7].

The XPS spectra attributed to O *1s* was characterized with three singlets at binding energies of 532.8, 531.5, and 529.8 eV, which correspond to Si–O, C=O, and Co oxide, respectively. As can be seen in Supplementary Fig. 13, the peaks assigned at 532.8 and 531.5 eV show strong intensity in the Pt_3Co_1 bimetallic NPs, whereas the peak signal associated with Co oxide (529.8 eV) is marginal. These results indicate that the O *1s* peak for Co oxide formed on the NP surface was buried due to the relatively large signal from the C=O bond in the organic capping layers (PVP) as well as the SiO_2 support (Si–O). This was further confirmed by the fact that the peak for Si–O increased and the peak for C=O decreased after the reaction. This tendency was the same in other NPs (Pt_1Co_1 and Pt_1Co_3). Exceptionally, in the case of Pt_0Co_1, where the size of the NPs is twice as large as the other NPs (*e.g.* Pt_3Co_1, Pt_1Co_1, Pt_1Co_3 NPs) and the NPs are deposited on SiO_2 substrate with higher coverage (~ 50 %), a relatively strong signal attributed to Co oxide (529.8 eV) was observed, implying that the metallic Co NPs are oxidized.

VB spectra of the Pt_xCo_y NPs were acquired to identify the electronic structure near the Fermi level and the oxidation states of the Co depending on the surface composition. As shown in Supplementary Fig. 14a, VB spectra of the PtCo bimetallic NPs have a broad peak near the Fermi level, which is distinguished from the electronic structures of metallic Co and Co_3O_4. Because the broad features appear from multiple splitting from unpaired 3d electrons and *p-d* charge transfer satellite splitting of CoO, it is clear that CoO exists on the PtCo NPs. We also observed that the density of states near the Fermi level decreased slightly with increasing Co, which implies that the higher the Co content, the greater the CoO, which is in agreement with the Co *2p* spectra. This tendency was confirmed more clearly by a rapid decrease in the electronic density of the PtCo bimetallic NPs after H_2 oxidation (Supplementary Fig. 14 b–f).

Supplementary Note 4 | *In situ* TEM measurement for defining the formation of CoO on bimetallic NPs. An aberration-corrected environmental TEM (Titan ETEM G2, FEI) operating at 300kV with a Fusion heating holder (Protochips inc.) was used for the *in situ* TEM observations. The Pt_3Co_1 NPs were supported on spherical silica particles with diameters of 380 nm and the Pt_3Co_1/SiO_2 sample was dispersed on a Protochip fusion E-chip that has no membranes in the viewing window and was inserted into the TEM. The Pt_3Co_1/SiO_2 sample was then heated from room temperature to 125 °C at a heating rate of 25 °C/min in 0.5 mbar of O_2 gas for 15 min inside the TEM. As shown in the sequential *in situ* TEM images (Supplementary Fig. 15) at 14 s, additional Co appeared on the top {111} surface of the Pt_3Co_1 NPs, as denoted by red arrows #1 and #2 in the image. The NP continued to undergo atom-by-atom growth along with the top {111} surface, where the newly grown layer was identified as a monolayer of CoO by a *d*-spacing of 2.46 Å which is consistent with that for CoO {111}. Despite further oxidation for 15 min, the monolayer of CoO remained without further deformation of the NPs (Supplementary Fig. 16).

Supplementary Note 5 | Theoretical calculations. Based on the XPS results for CoO formation on Pt, we modelled a CoO island on the Pt (111) surface (Supplementary Fig.17). H_2 oxidation consists of the following four intermediate reaction steps (i) ~ (iv)[8, 9]:

$$*O_2 \rightarrow 2*O \qquad (i)$$

$$*H_2 \rightarrow 2*H \qquad (ii)$$

$$*O + *H \rightarrow *OH \qquad (iii)$$

$$*OH + *H \rightarrow *H_2O \qquad (iv)$$

where * indicates adsorbed species on the catalyst surface. For a pure Pt (111) surface, it was reported that reaction steps (i), (ii), and (iv) are kinetically facile with activation barriers of 0.2[10], 0.1[11], and 0.2 eV[8], respectively. On the other hand, reaction step (iii) is sluggish with an activation barrier of approximately 0.9 eV, making it the rate-determining step[8]. Thus, in the case of CoO/Pt, one can also expect that the O_2 and H_2 dissociation reactions (*i.e.* steps (i) and (ii)) occur rapidly and predominantly on the Pt portion of the surface. However, the CoO/Pt interface may play a key role in lowering the activation barrier for OH formation (*i.e.* step (iii))[8-10], in contrast to Pt (111).

We compared the activation barriers for OH formation on Pt (111) and at the CoO/Pt interface sites. The calculated activation barriers are 0.88 and 0.72 eV for Pt (111) (Fig. 5a) and the CoO/Pt interface (Fig. 5b), respectively; indeed, the interfacial sites are more active than the bulk Pt sites by 0.16 eV. To understand the origin of the lower activation barrier at the interface, we decomposed the O–H bond formation into three steps (Supplementary Fig. 18 and Table 3): (A) Pt–O bond rearrangement (0.59 vs. 0.37 eV for Pt (111) and CoO/Pt, respectively), (B) H migration (0.37 vs. 0.46 eV), and (C) stabilization by the O–H bond (−0.08 vs. −0.11 eV). As can be seen in this brief analysis, Pt–O bond rearrangements are the major bottleneck for the OH formation reaction for Pt (111), but for CoO/Pt, the Pt–O bond rearrangement becomes much more facile by 0.22 eV compared with Pt (111). The origin of this difference lies in the fact that, on the Pt (111) surface, *O moves from a fcc-hollow site at the initial state to a bridge site at the transition state, traveling 0.95 Å; however, at the CoO/Pt interface, *O remains at the top site of Pt (111), moving only 0.26 Å.

We next investigated H_2O formation via direct association of *OH with *H, reaction step (iv), for Pt (111) and the CoO/Pt interface; the calculated barriers were 0.25 and 0.74 eV, respectively (Supplementary Fig. 19), where the activation needed for H_2O formation for Pt (111) is consistent with that found in the literature[8]. Although the direct association of *OH and *H at the CoO/Pt interfacial sites requires a substantially larger barrier than for the Pt (111) case, the barrier is still comparable to that of *O–*H association at the CoO/Pt interface. Alternatively, we investigated a H-migration pathway that bypasses the energy-intensive direct *OH + *H association step at the CoO/Pt interface. In this pathway, *O on the Pt domain migrates to the periphery of the CoO/Pt interface, followed by H-migration from the CoO to the nearby *O sites on the Pt, and undergoes the usual low-barrier $*H_2O$ formation reaction in the Pt region, as illustrated in Fig. 5c. The activation barriers for each intermediate reaction step described above are indeed all calculated to be small (*i.e.* 0.29, 0.15, and 0.25 eV, respectively). Thus, both direct association and H-migration pathways indicate that the CoO/Pt interface is more active for H_2 oxidation than Pt (111).

Supplementary Figures

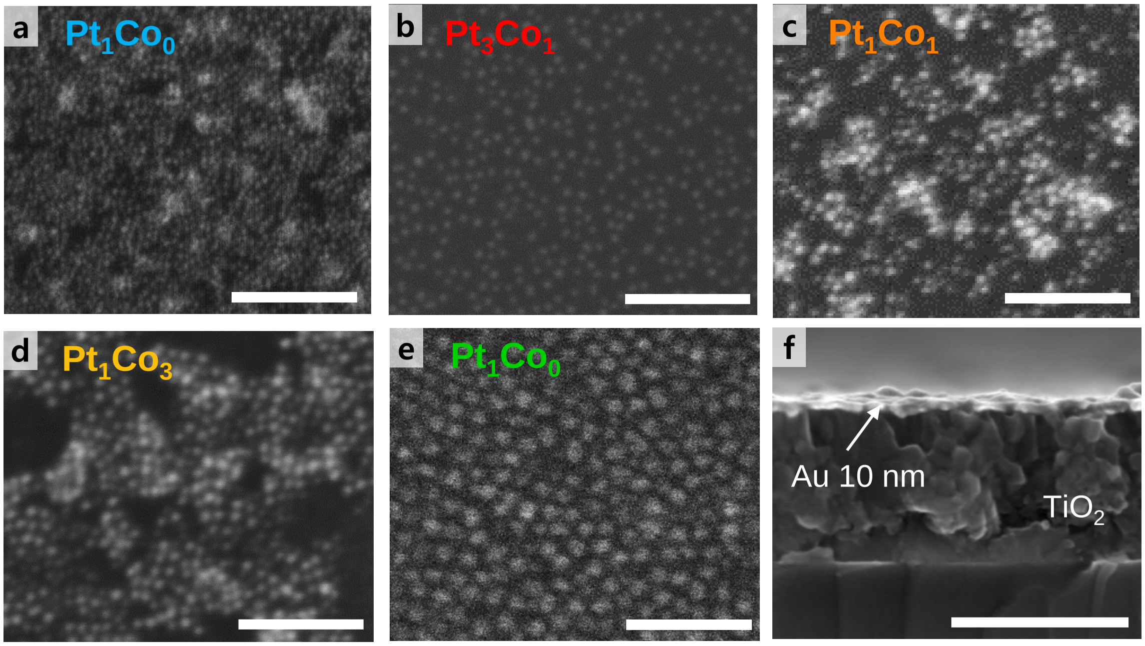

Supplementary Figure 1 | SEM images of Langmuit–Blodgett films of Pt_xCo_y bimetallic NPs with different compositions: **a** Pt_1Co_0, **b** Pt_3Co_1, **c** Pt_1Co_1, **d** Pt_1Co_3, and **e** Pt_0Co_1. Scale bars are 50 nm. (f) Cross-sectional field emission SEM image of a 10 nm Au film on a 250 nm TiO_2 layer. Scale bar is 300 nm.

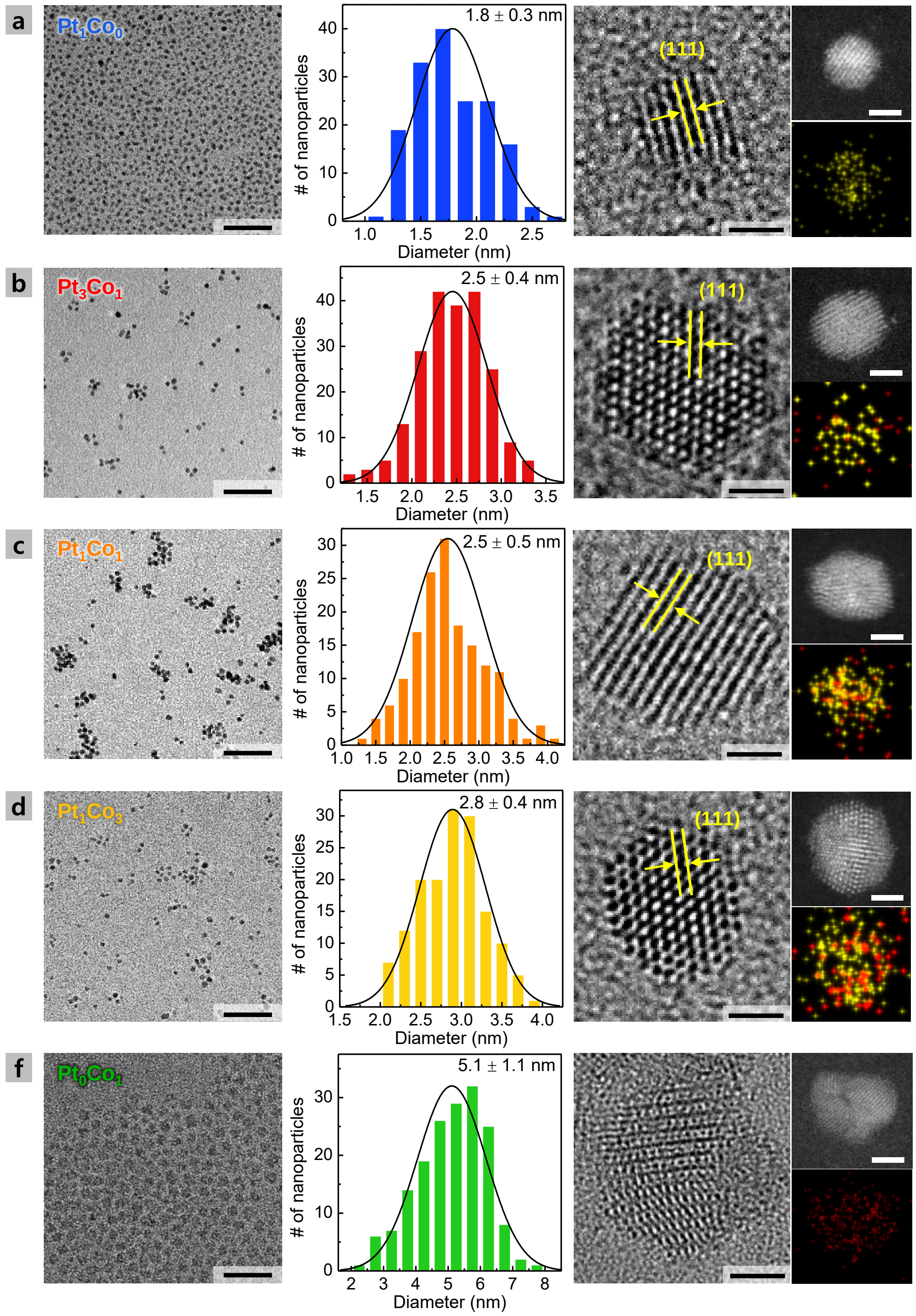

Supplementary Figure 2 | TEM, size distribution histogram, high-resolution TEM (HRTEM) images, HAADF-STEM images, and STEM-EDS mappings of as-synthesized **a** Pt_1Co_0, **b** Pt_3Co_1, **c** Pt_1Co_1, **d** Pt_1Co_3, and **e** Pt_0Co_1 bimetallic NPs. Elemental mapping was performed by acquiring EDS images, including the Co K series (red) and Pt L (yellow). Scale bars are 20 nm (left), 1 nm (middle), and 1 nm (right) (a). Scale bars are 30 nm (left), 1 nm (middle), and 1 nm (right) (b –e). Scale bars are 20 nm (left), 2 nm (middle), and 2 nm (right) (d).

a

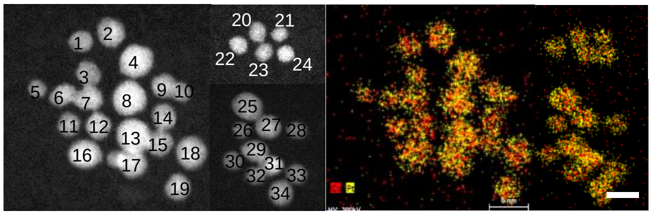

b

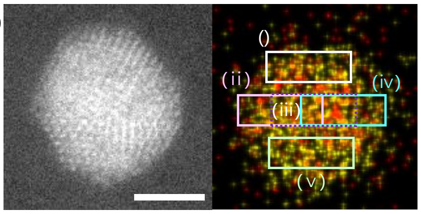

	Atomic %			Atomic %	
	Pt-L	Co-K		Pt-L	Co-K
1	72.36	27.64	18	78.62	21.38
2	74.44	25.56	19	84.09	15.91
3	75.82	24.18	20	78.73	21.27
4	74.01	25.99	21	86.70	13.30
5	79.73	20.27	22	82.46	17.54
6	75.96	24.04	23	83.39	16.61
7	69.11	30.89	24	77.38	22.62
8	62.00	38.00	25	79.52	20.48
9	79.26	20.74	26	77.83	22.17
10	80.75	19.25	27	80.70	19.30
11	78.05	21.95	28	74.44	25.56
12	76.71	23.29	29	78.82	21.18
13	78.17	21.83	30	76.64	23.36
14	82.75	17.25	31	77.69	22.31
15	73.30	26.70	32	80.91	19.09
16	76.19	23.81	33	83.21	16.79
17	76.08	23.92	34	76.29	23.71
**Pt % : Co % = 77.7 ± 4.4 : 22.3 ± 4.4**					

	Atomic %	
	Pt-L	Co-K
(i)	73.10	26.90
(ii)	81.90	18.10
(iii)	75.60	24.40
(iv)	74.60	25.40
(v)	81.20	18.80
**Pt % : Co % = 77.3 ± 3.3 : 22.7 ± 3.3**		

Supplementary Figure 3 | a HAADF-STEM images and STEM-EDS mapping images with elemental analysis for individual Pt_3Co_1 NPs as labelled by numbers. Scale bar is 5 nm. **b** Area scan analysis in different regions of the Pt_3Co_1 NP, confirming the homogeneous atomic distribution of the bimetallic NP. Scale bar is 2 nm.

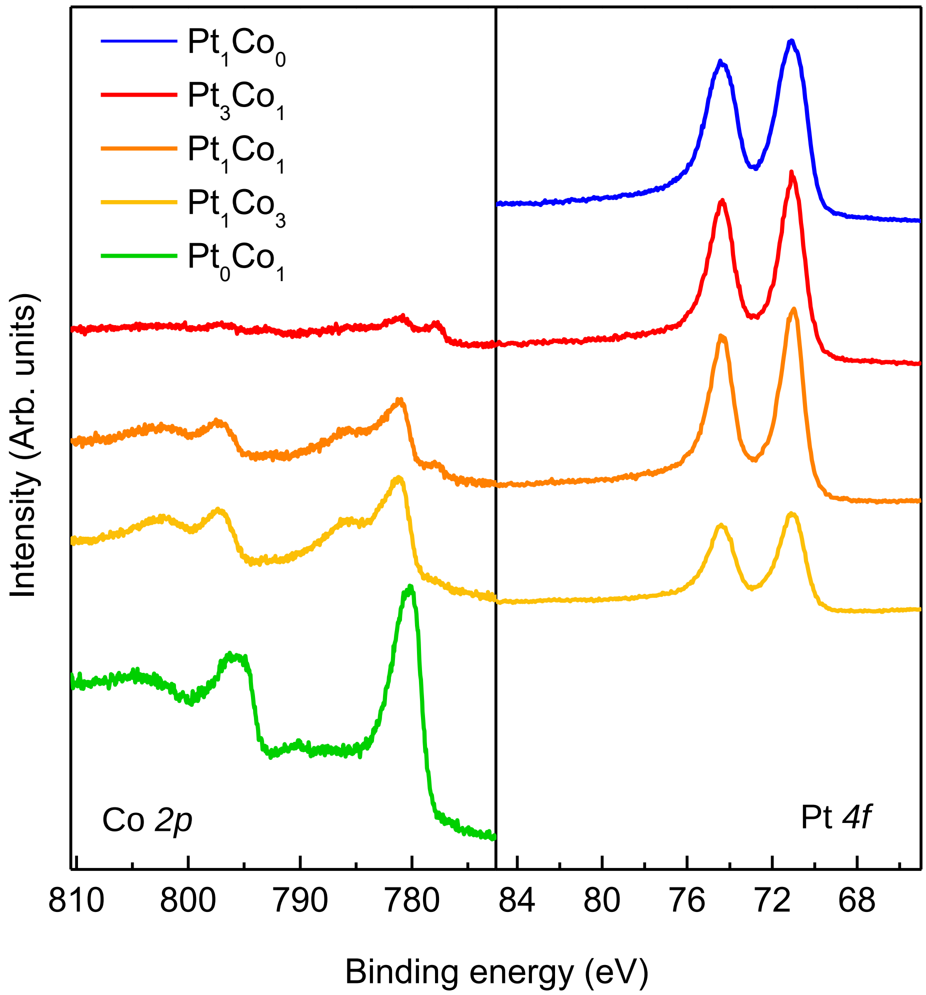

Supplementary Figure 4 | Chemical composition analysis using XPS. Co *2p* and Pt *4f* XPS spectra of as-synthesized Pt_xCo_y bimetallic NPs of different compositions: Pt_1Co_0 (blue), Pt_3Co_1 (red), Pt_1Co_1 (orange), Pt_1Co_3 (yellow), and Pt_0Co_1 (green) NPs.

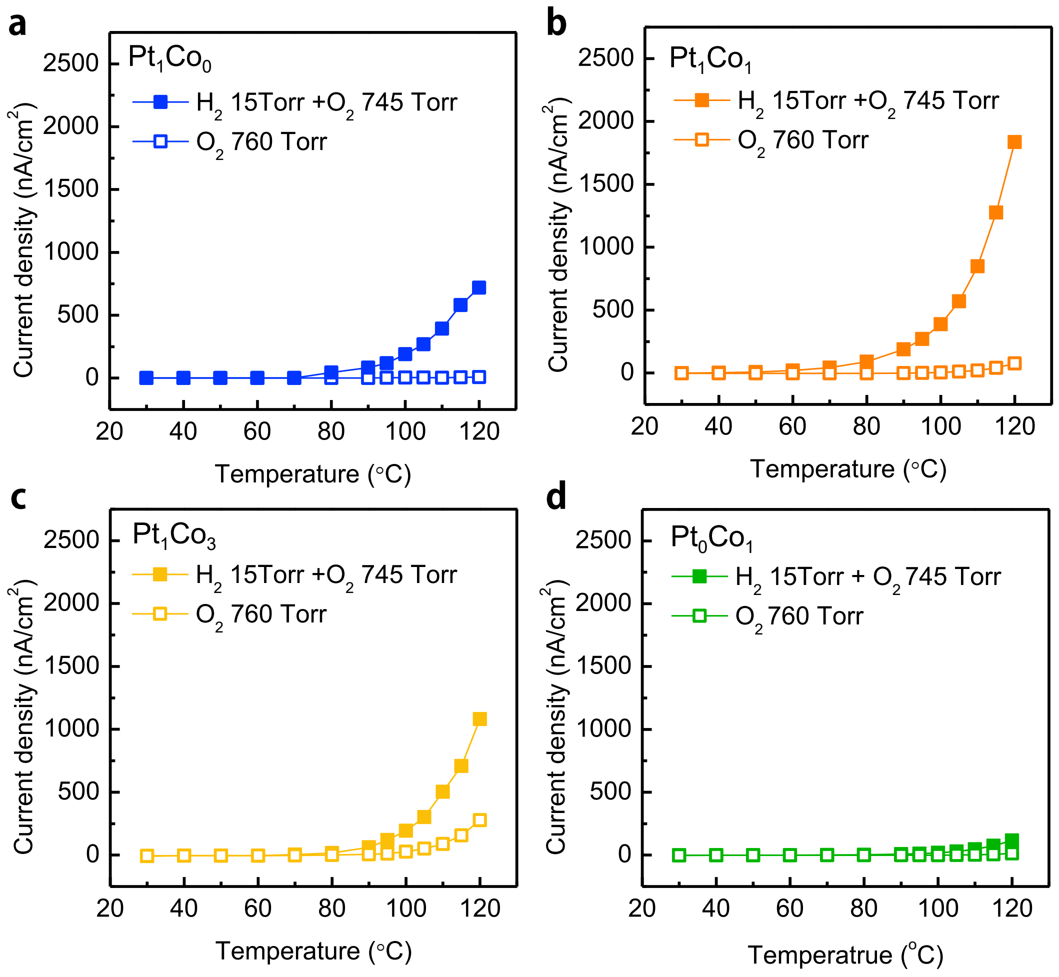

Supplementary Figure 5 | Temperature dependence of the current from Au/TiO_2 nanodiodes with **a** Pt_1Co_0, **b** Pt_1Co_1, **c** Pt_1Co_3, and **d** Pt_0Co_1 NPs measured in the H_2 + O_2 gas mixture and in pure O_2. The current signals measured during the catalytic reaction in the H_2 + O_2 mixture deviate significantly from the current measured under the O_2 condition, except in the case of bare Co NPs.

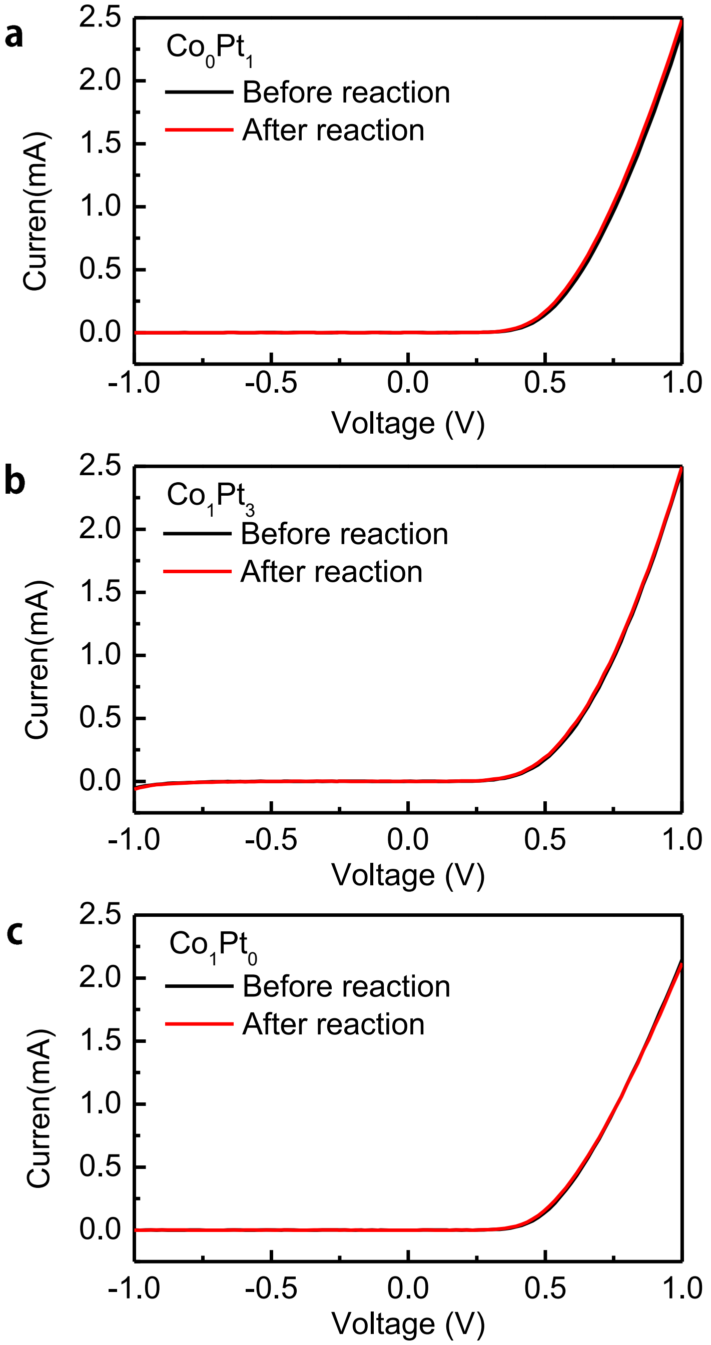

Supplementary Figure 6 | Typical I–V curves of Au/TiO_2 nanodiodes composed of **a** Pt_1Co_0, **b** Pt_3Co_1, and **c** Pt_0Co_1 NPs on a 10 nm Au film, before and after reaction. The properties of the diode remained constant without a significant change in the Schottky barrier height even though the samples were heated up to 120 °C during the chemicurrent measurements.

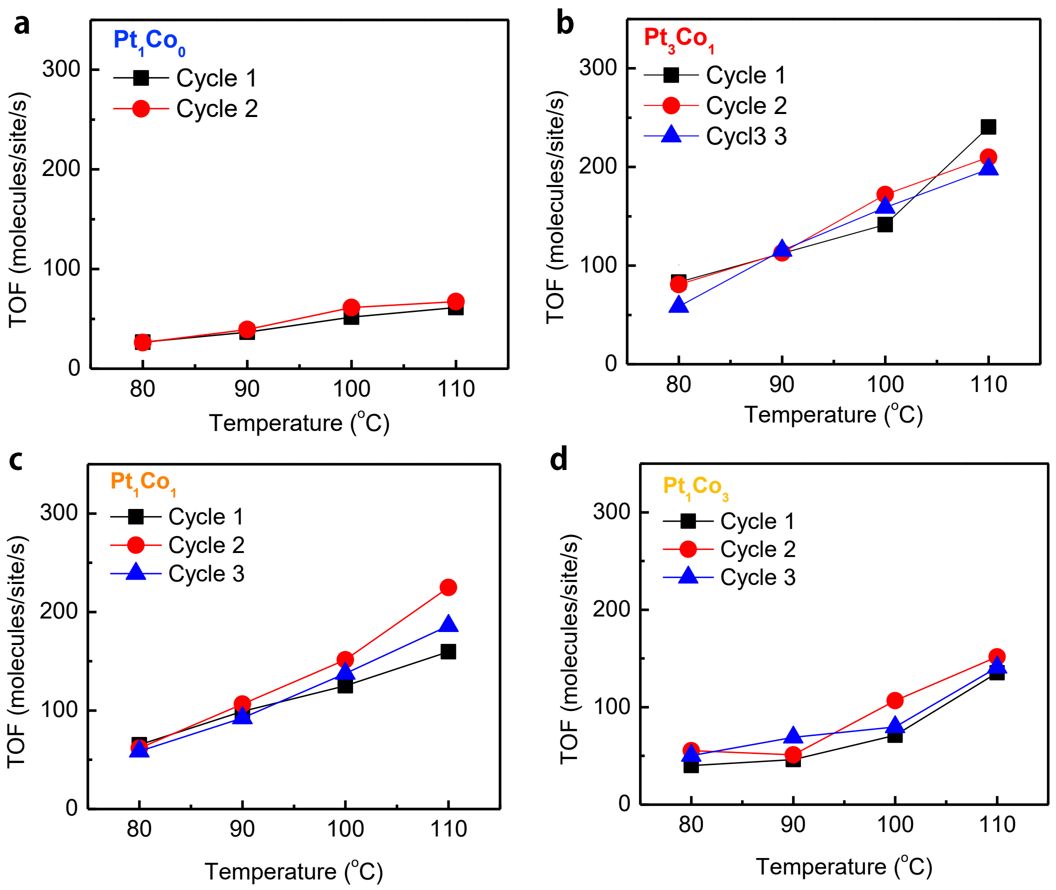

Supplementary Figure 7 | Reproducibility of the catalytic activity for **a** Pt_1Co_0, **b** Pt_3Co_1, **c** Pt_1Co_1, and **d** Pt_1Co_3 NPs, when the TOF measurements were repeated 2–3 times. The catalytic performance of the NPs is reproducible.

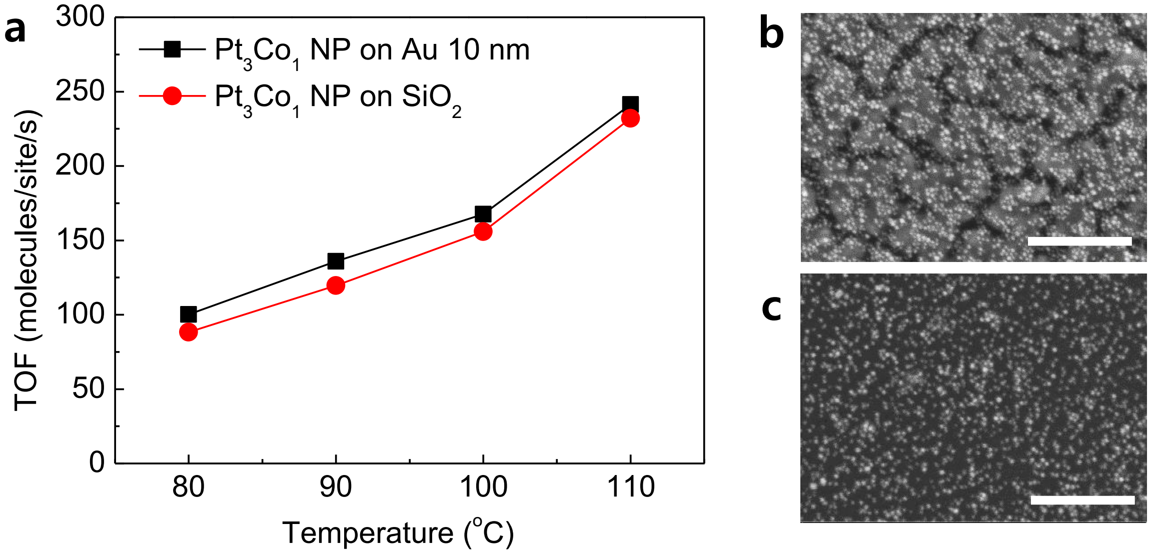

Supplementary Figure 8 | a The catalytic activity for H_2 oxidation of Pt_3Co_1 bimetallic NPs supported on 10 nm Au film/SiO_2 (black line) and SiO_2 substrate (red line) in the temperature range of 80 °C – 110 °C. SEM images of Langmuir–Blodgett films of Pt_3Co_1 bimetallic NPs deposited on **b** 10 nm Au film/SiO_2 and **c** SiO_2 substrate. Scale bars are 100 nm.

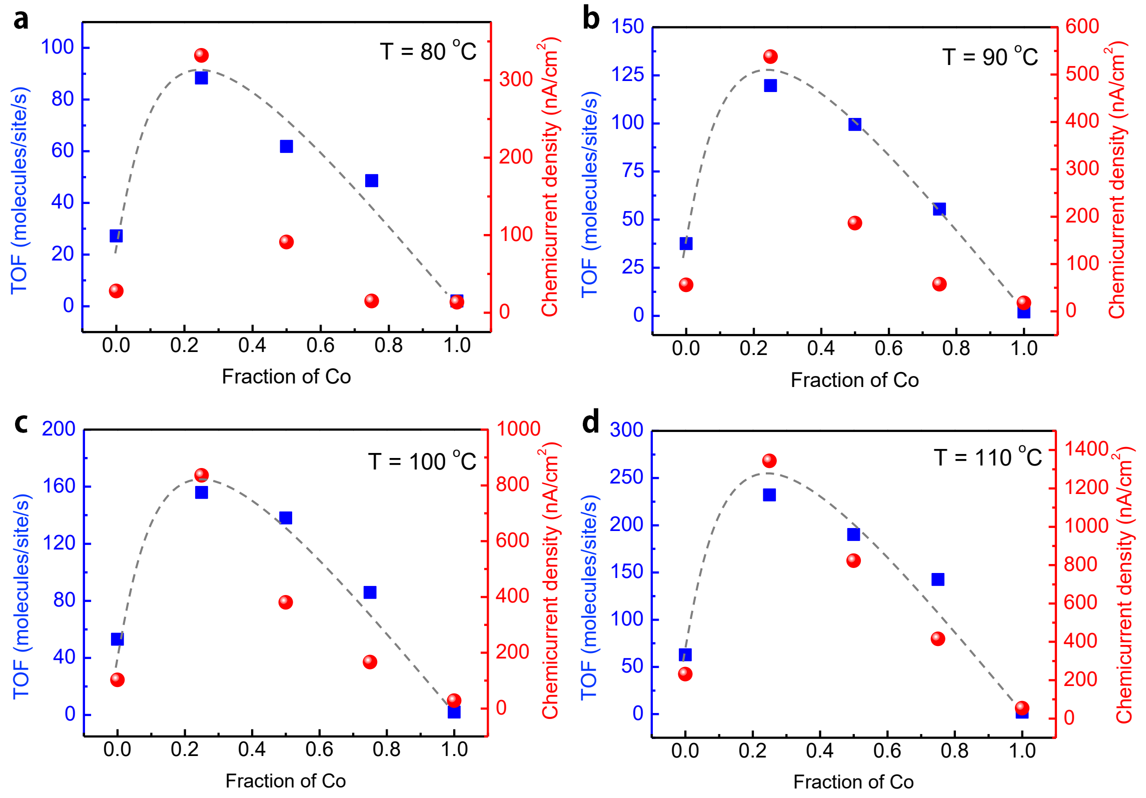

Supplementary Figure 9 | Plot comparing both chemicurrent and TOF as a function of the composition of the PtCo bimetallic NPs at **a** 80 °C, **b** 90 °C, **c** 100 °C, and **d** 110 °C.

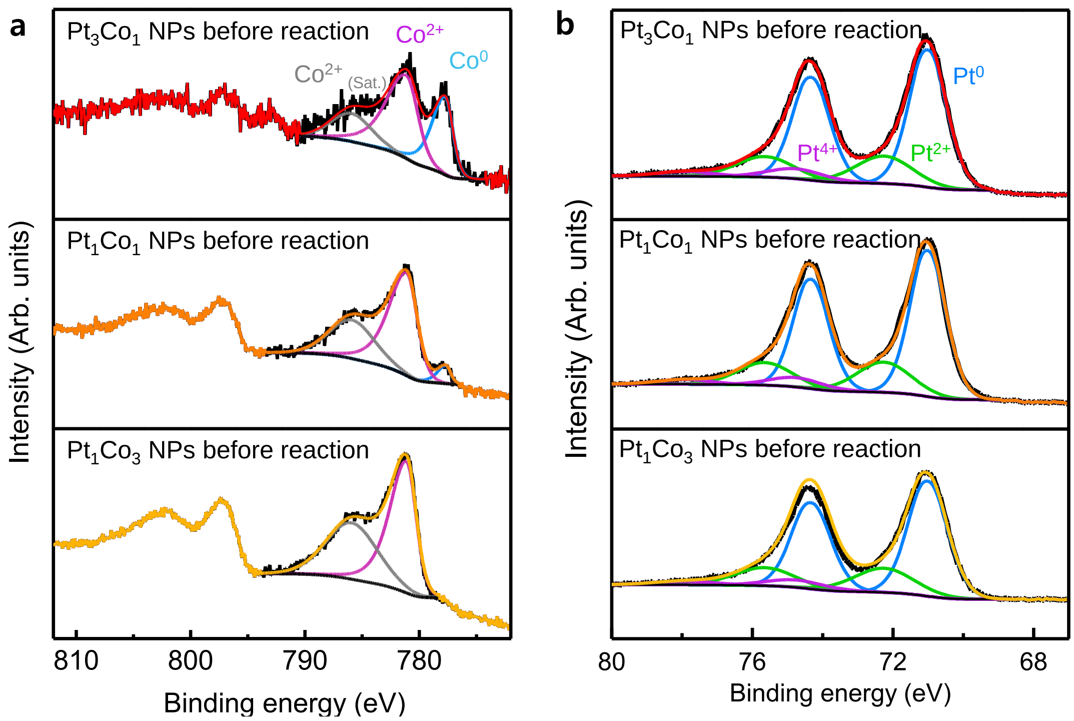

Supplementary Figure 10 | Oxidation states of the elements in the Pt_xCo_y bimetallic NPs. **a** Co *2p* and **b** Pt *4f* XPS spectra of the as-synthesized Pt_3Co_1 (red), Pt_1Co_1 (orange), and Pt_1Co_3 (yellow) bimetallic NPs.

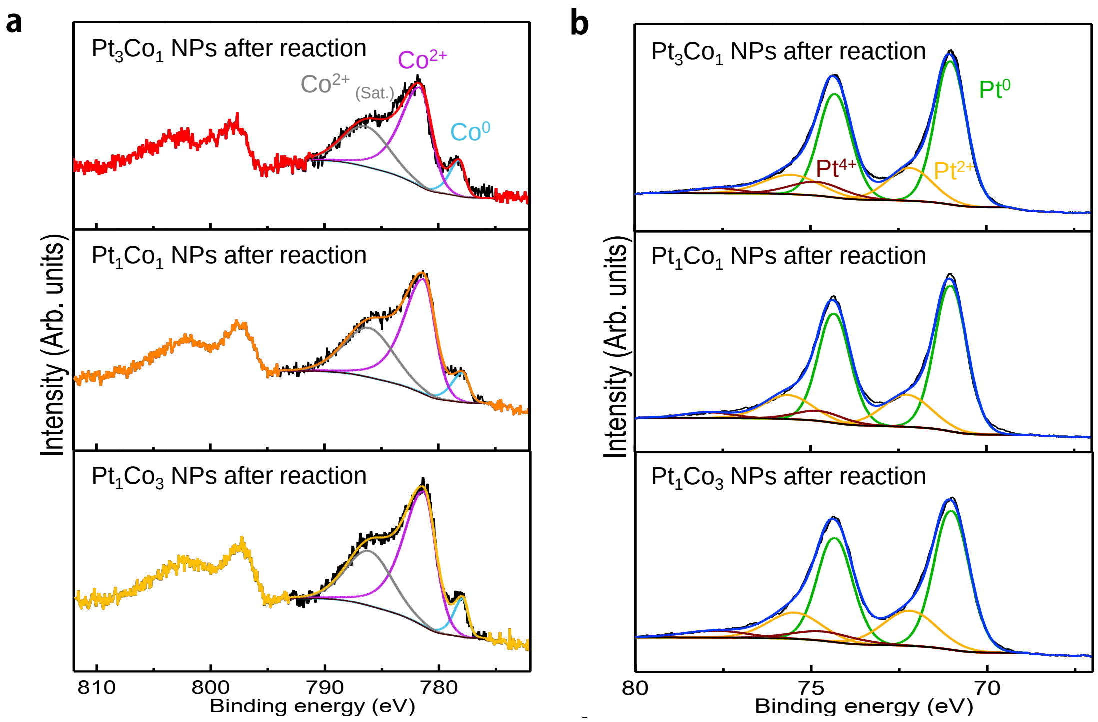

Supplementary Figure 11 | Oxidation states of the elements in the Pt_xCo_y bimetallic NPs. **a** Co *2p* and **b** Pt *4f* XPS spectra after the reaction of Pt_3Co_1, Pt_1Co_1, and Pt_1Co_3 bimetallic NPs

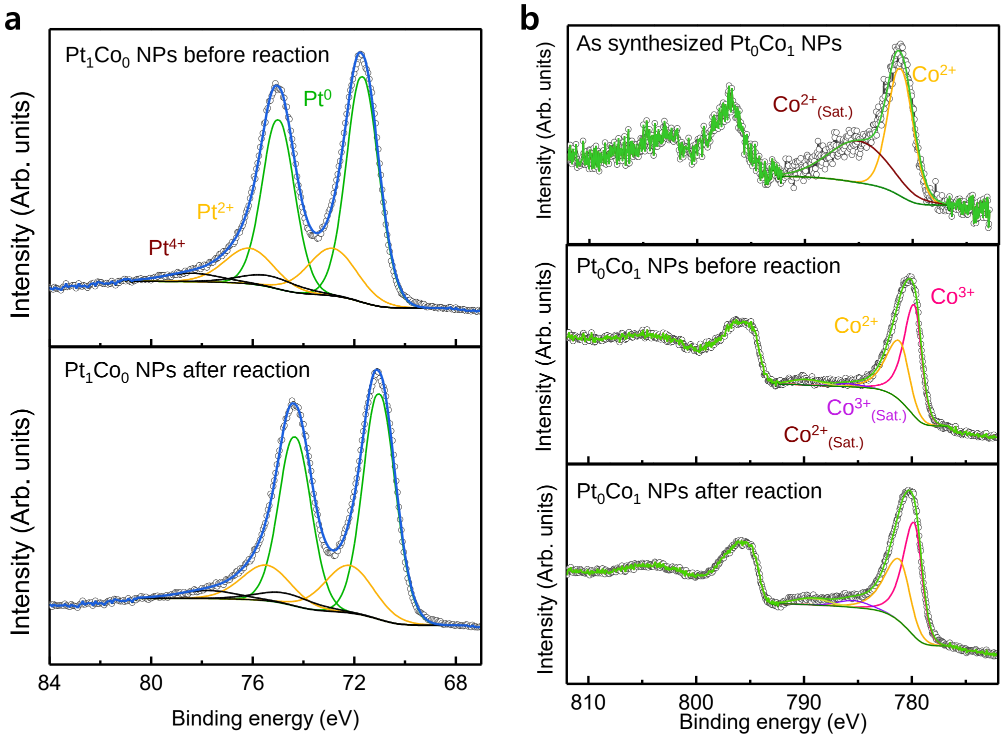

Supplementary Figure 12 | a Pt *4f* XPS spectra of the Pt_1Co_0 NPs before and after reaction. **b** Co *2p* XPS spectra of the as synthesized Pt_0Co_1 NPs and before and after reaction.

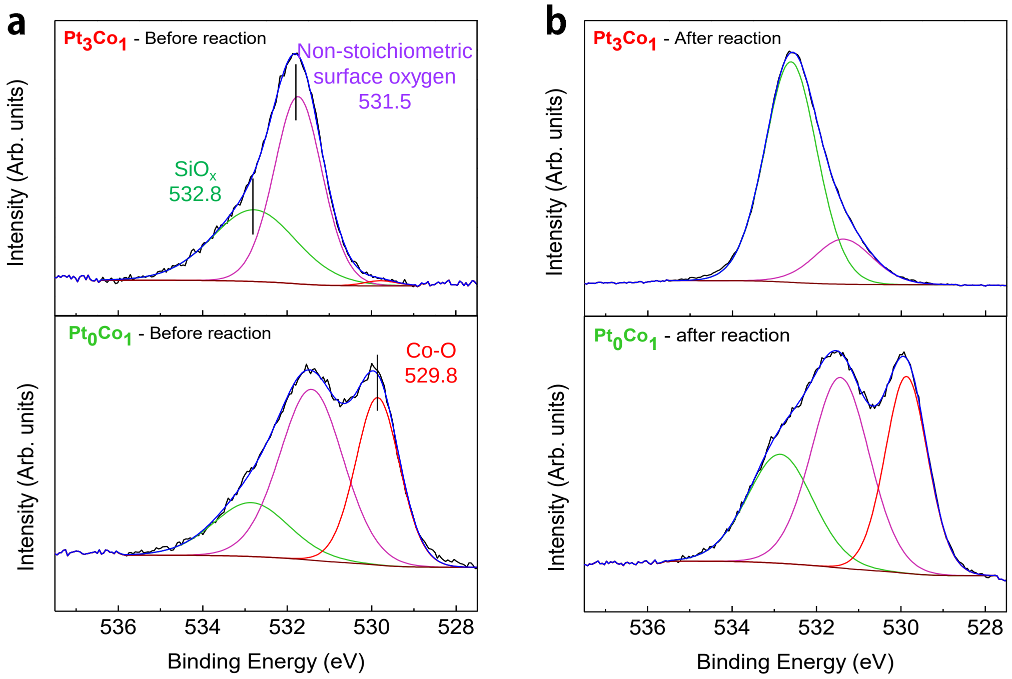

Supplementary Figure 13 | O *1s* XPS spectra of Pt_3Co_1 and Pt_0Co_1NPs **a** before and **b** after reaction.

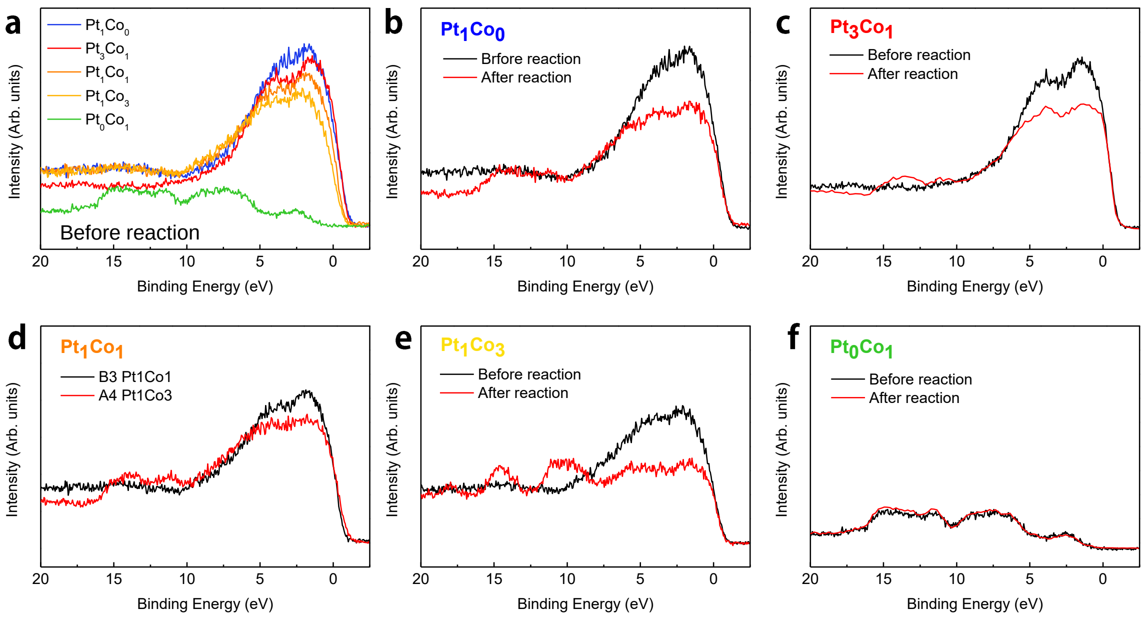

Supplementary Figure 14 | a Valence band (VB) XPS spectra of Pt_xCo_y bimetallic NPs of different compositions: Pt_1Co_0 (blue), Pt_3Co_1 (red), Pt_1Co_1 (orange), Pt_1Co_3 (yellow), and Pt_0Co_1 (green) NPs. **b – f** Comparison of VB spectra of Pt_xCo_y NPs before and after reaction.

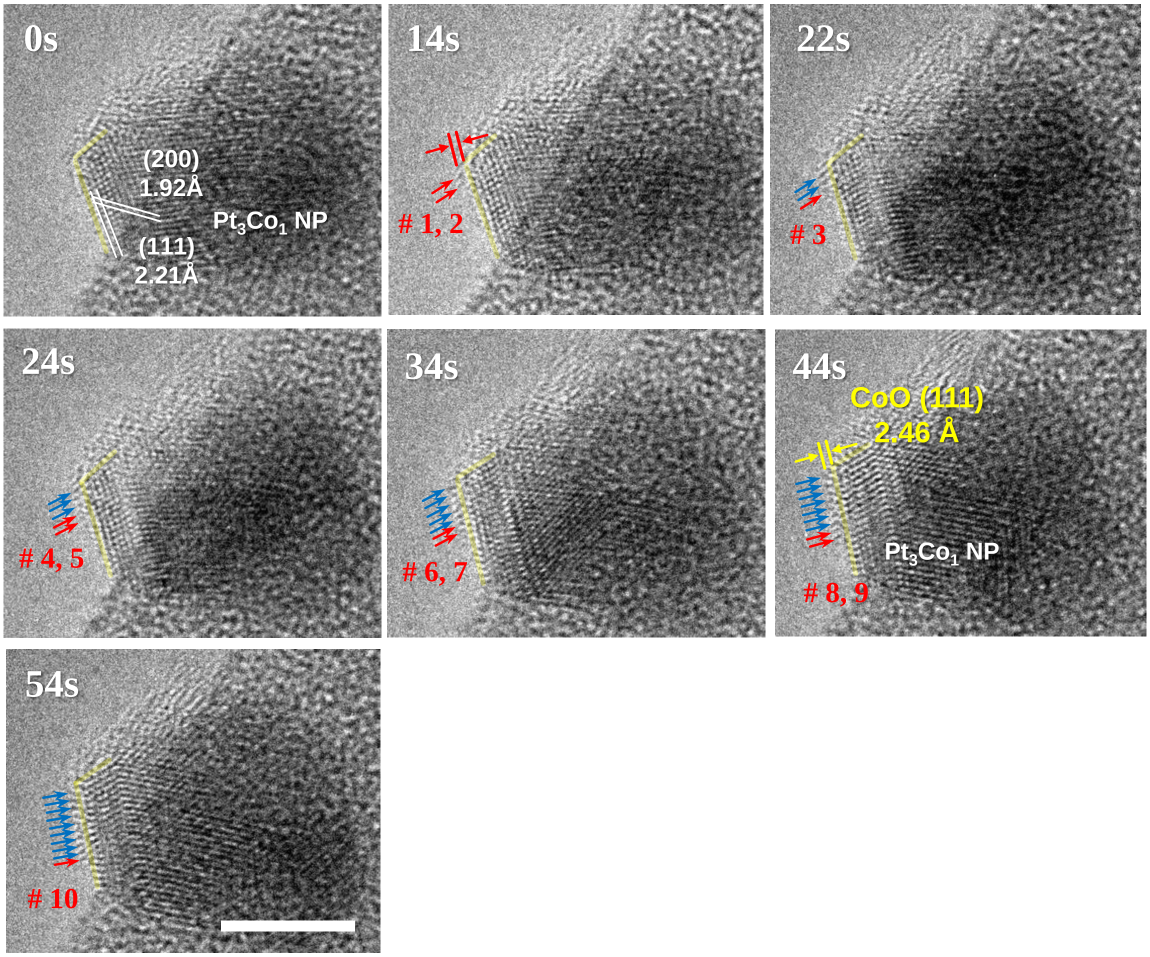

Supplementary Figure 15 | Sequential TEM images of Pt_3Co_1 NPs during oxidization at 125 °C in 0.5 mbar of O_2 gas environment. Scale bar is 5 nm.

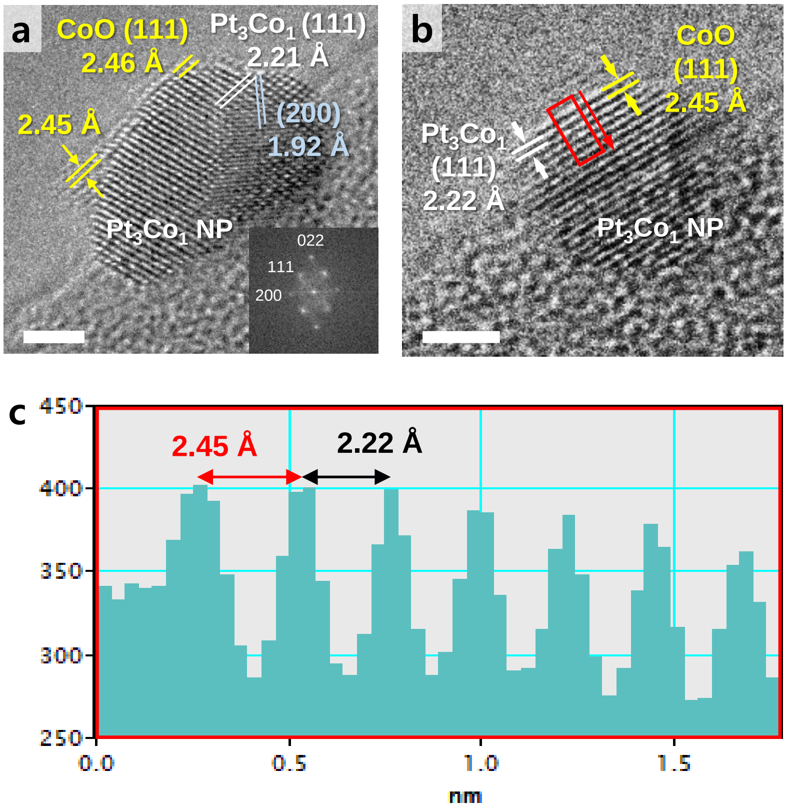

Supplementary Figure 16 | **a** TEM image showing the formation of the CoO layer on the surface of the oxidized Pt_3Co_1 NPs at 125 °C for 15 min (0.5 mbar O_2 gas environment). Corresponding fast Fourier transform pattern illustrates the fcc structure of the alloyed Pt_3Co_1 NPs. **b** Another example of CoO formation on the surface of oxidized Pt_3Co_1 NPs at 125 °C for 15 min. Scale bars are 2 nm (a, b). **c** Intensity profile taken along the arrow in the red rectangle in (b).

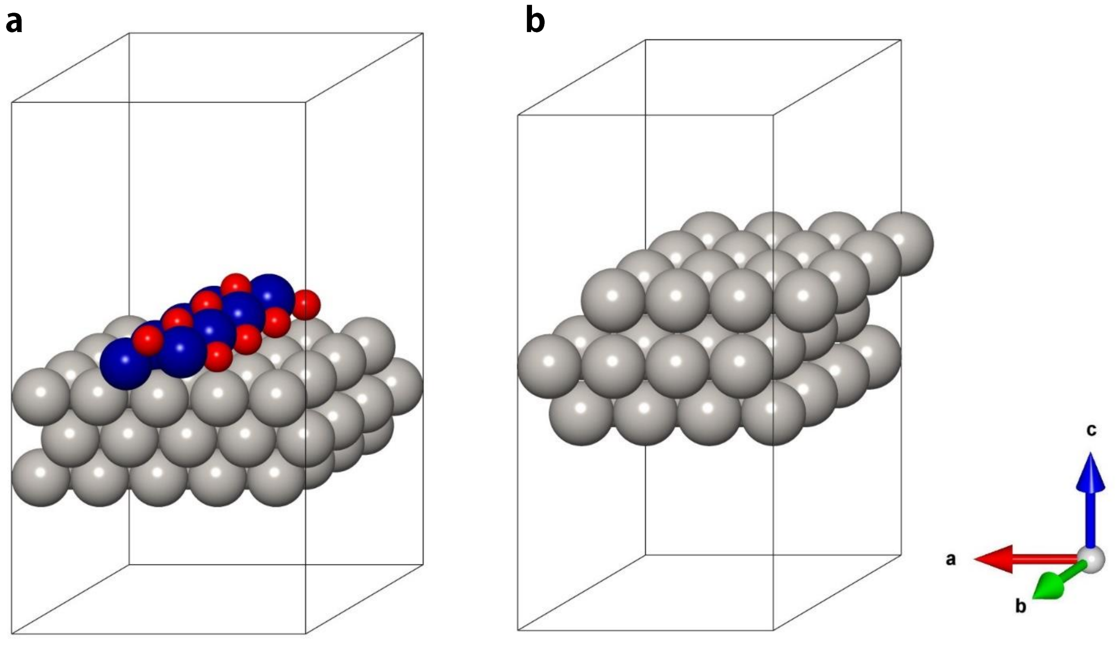

Supplementary Figure 17 | Computational unit cell of **a** a CoO island on Pt (111) and **b** Pt (111).

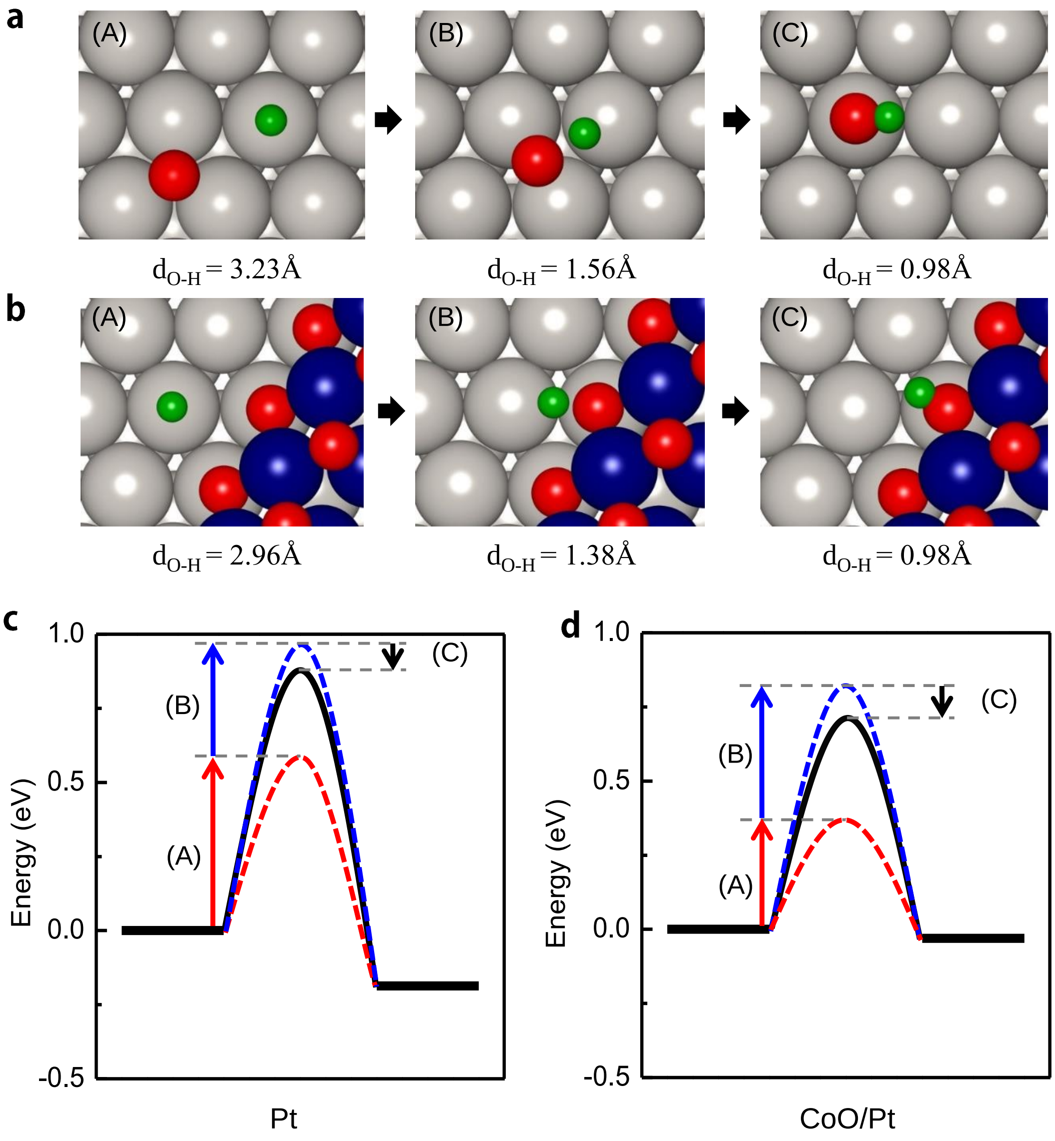

Supplementary Figure 18 | Images for initial, transition, and final states of the *OH formation pathway **a** on the Pt (111) surface and **b** at the CoO/Pt interface. Energy decomposition analysis for the *OH formation step **c** on Pt (111) and **d** at the CoO/Pt interface. The activation energy is decomposed into three steps: (A) Pt–O bond arrangement, (B) H migration, and (C) stabilization by the O–H bond. All values are summarized in supplementary Table 3.

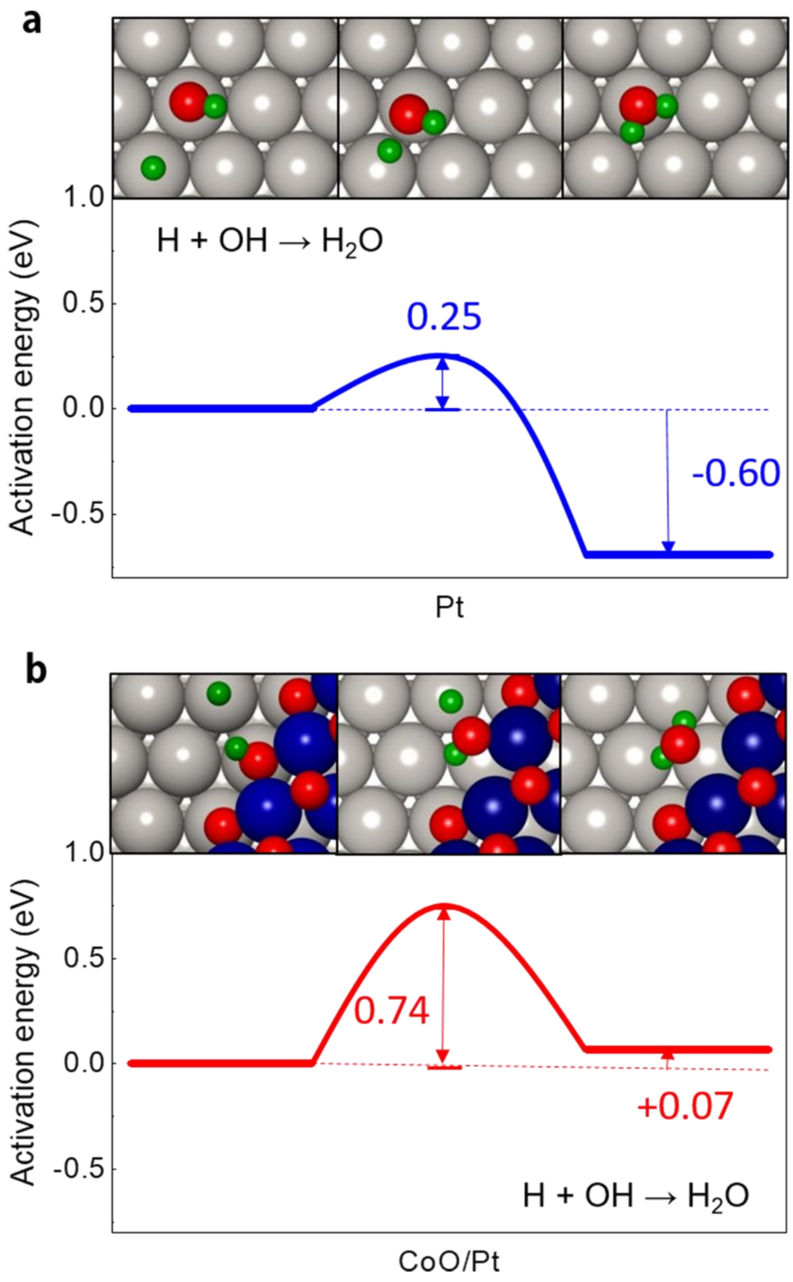

Supplementary Figure 19 | Energy diagram of the $*OH_2$ formation pathway **a** on Pt (111) and **b** at the periphery of the CoO/Pt interface.

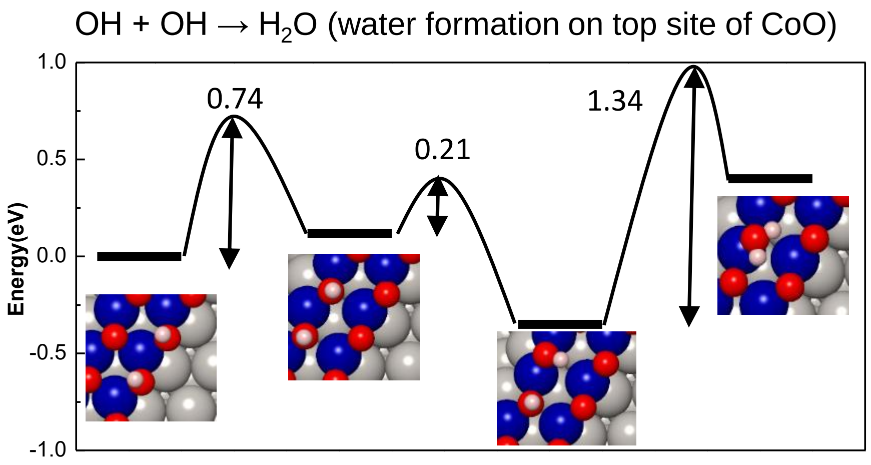

Supplementary Figure 20 | Energy diagram of the *OH_2 formation pathway on the top site of the CoO monolayer

Supplementary Tables

	Target composition (at %)		Composition by XPS (at %)		Composition by ICP-MS (at %)	
	Co	Pt	Co	Pt	Co	Pt
Pt_1Co_0	0	100	0	100	0	100
Pt_3Co_1	25	75	19.5	80.5	35.8	64.2
Pt_1Co_1	50	50	44.6	55.4	47.6	52.4
Pt_1Co_3	75	25	68.3	31.7	83.5	16.5
Pt_0Co_1	100	0	100	0	100	0

Supplementary Table 1 | Comparison of composition values of Pt_xCo_y bimetallic NPs (x:y = 1:0, 3:1, 1:1, 1:3, 0:1) obtained from XPS and ICP-MS analysis.

	Nanoparticle size (nm)	
	TEM	XRD
Pt_1Co_0	1.8 ± 0.3	1.8 ± 0.1
Pt_3Co_1	2.5 ± 0.4	2.4 ± 0.1
Pt_1Co_1	2.5 ± 0.5	2.4 ± 0.1
Pt_1Co_3	2.8 ± 0.4	2.1 ± 0.1
Pt_0Co_1	5.1 ± 1.1	5.3 ± 0.5

Supplementary Table 2 | Comparison of size for the five NPs (Pt_1Co_0, Pt_3Co_1, Pt_1Co_1, Pt_1Co_3, and Pt_0Co_1) estimated by TEM and XRD.

	Pt-O bond rearrangement<br><br>(A)	H migration<br><br>(B)	Stabilization by OH bond<br><br>(C)	Net energy barrier<br><br>(A+B+C)
Pt	0.59	0.38	-0.08	0.88
CoO/Pt	0.37	0.45	-0.11	0.72

Supplementary Table 3 | Decomposed energies for *OH formation. The values are obtained by calculating the energy difference between the initial and transition states of the reactant. The single point calculation is performed to get the energy difference of removing a (A) H atom and/or (B) O atom for the initial and transition states. (C) The difference between the activation energy and the combined value of (A) and (B) indicates stabilization by the OH bond at the transition state. All values are given in eV.

Supplementary References

1. An, K. & Somorjai, G. A. Size and shape control of metal nanoparticles for reaction selectivity in catalysis. *ChemCatChem* **4**, 1512-1524 (2012).
2. Yu, W., Wang, Y., Liu, H. & Zheng, W. Preparation and characterization of polymer-protected PtCo bimetallic colloids and their catalytic properties in the selective hydrogenation of cinnamaldehyde. *J. Mol. Catal. A: Chem.* **112**, 105-113 (1996).
3. Papaefthimiou, V. *et al.* When a metastable oxide stabilizes at the nanoscale: wurtzite CoO formation upon dealloying of PtCo nanoparticles. *J. Phys. Chem. Lett.* **2**, 900-904 (2011).
4. Xin, H. L. *et al.* Revealing the atomic restructuring of Pt–Co nanoparticles. *Nano Lett.* **14**, 3203-3207 (2014).
5. Fu , Q. *et al.* Interface-confined ferrous centers for catalytic oxidation. *Science* **328**, 1141-1144 (2010).
6. Ning, Y. *et al.* Nature of Interface Confinement Effect in Oxide/Metal Catalysts. *J. Phys. Chem. C* **119**, 27556-27561 (2015).
7. Xu, H., Fu, Q., Guo, X. & Bao, X. Architecture of Pt-Co bimetallic catalysts for catalytic CO Oxidation. *ChemCatChem* **4**, 1645-1652 (2012).
8. Michaelides, A. & Hu, P. Catalytic water formation on platinum: A first-principles study. *J. Am. Chem. Soc.* **123**, 4235-4242 (2001).
9. Hellsing, B., Kasemo, B. & Zhdanov, V. Kinetics of the hydrogen-oxygen reaction on platinum. *J. Catal.* **132**, 210-228 (1991).
10. Li, R., Li, H. & Liu, J. First principles study of O_2 dissociation on Pt (111) surface: Stepwise mechanism. *Int. J. Quantum Chem*, **116**, 908-914 (2016).
11. Arboleda Jr, N. B., Kasai, H., Dino, W. A. & Nakanishi, H. Potential energy of H_2 dissociation and adsorption on Pt (111) surface: first-principles calculation. *Jpn. J. Appl. Phys.* **46**, 4233 (2007).
