# Supplementary material for: Boosting hot electron flux and catalytic activity at metal–oxide interfaces of PtCo bimetallic nanoparticles
Source: Nat Commun. 2018 Jun 8;9:2235. doi: 10.1038/s41467-018-04713-8 (PMC5993833; doi:10.1038/s41467-018-04713-8)
Supplement: Supplementary file 2 — Descriptions of Additional Supplementary Files [file 41467_2018_4713_MOESM2_ESM.pdf]

Descriptions for Additional Supplementary Files

File Name: Supplementary Movie 1
Description: Sequence of in-situ transmission electron microscopy images of Pt3Co1 nanoparticles at 125 °C and oxygen gas condition.
